# Diagnosis of Metal Hypersensitivity in Total Knee Arthroplasty: A Case Report

**DOI:** 10.3389/fimmu.2019.02758

**Published:** 2019-11-27

**Authors:** Janosch Schoon, Melanie J. Ort, Katrin Huesker, Sven Geissler, Anastasia Rakow

**Affiliations:** ^1^Julius Wolff Institute, Charité-Universitätsmedizin Berlin, Corporate Member of Freie Universität Berlin, Humboldt-Universität zu Berlin, Berlin Institute of Health, Berlin, Germany; ^2^Berlin Institute of Health Center for Regenerative Therapies, Berlin, Germany; ^3^Berlin-Brandenburg School for Regenerative Therapies, Charité-Universitätsmedizin Berlin, corporate member of Freie Universität Berlin, Humboldt-Universität zu Berlin, Berlin Institute of Health, Berlin, Germany; ^4^Immunology Department, Institute for Medical Diagnostics, Berlin, Germany; ^5^Center for Musculoskeletal Surgery, Charité-Universitätsmedizin Berlin, corporate member of Freie Universität Berlin, Humboldt-Universität zu Berlin, Berlin Institute of Health, Berlin, Germany

**Keywords:** delayed type hypersensitivity, arthroplasty, lymphocyte transformation test, T cell subsets, allergy diagnostics

## Abstract

Delayed type hypersensitivity (DTH) reactions are considered infrequent complications in arthroplasty, but have been recognized to be associated with devastating morbidity and substantial decrease in quality of life of affected patients. Chronic inflammation of artificial joints and associated loss of peri-implant bone often require revision surgery. Methods for the diagnosis of implant-related DTH are available but infrequently considered to the full extent. Sequential diagnostics based on exclusion of septic complications, local and systemic metal level determination, lymphocyte transformation testing (LTT), and local T cell subset analysis are required for an unequivocal DTH diagnosis. Here, we report on a patient with a history of chronic rheumatoid arthritis and an unfavorable outcome of unilateral knee arthroplasty. This case illustrates pitfalls and difficulties in the course of recurrent inflammation following joint replacement. In the early course, suspicion of low-grade bacterial infection led to three two-stage revisions. Afterwards, the joint was proven to be sterile. However, metal level quantification revealed release of especially cobalt and chromium from the joint, LTT indicated persisting cobalt and nickel sensitization and subset analysis of T cells from the synovium suggested DTH as a root cause for the inflammatory symptoms. This report aims to recommend the depicted diagnostic algorithm as an adequate tool for future DTH detection. Yet, systemic to local subset ratios for effector memory and regulatory T cells should be derived from sufficient patient numbers to establish it as a diagnostic marker. Moreover, future prospects regarding implant-related DTH diagnostics are discussed. Therapeutic options for the portrayed patient are proposed, considering pharmaceutical, cell-therapeutic and surgical aspects. Patients who experience peri-implant inflammation but do not have obvious mechanical or infectious problems remain a diagnostic challenge and are at high risk of being treated inadequately. Since potentially sensitizing materials are regularly used in arthroplasty, it is essential to detect cases of acute DTH-derived inflammation of an artificial joint at early postoperative stages. This would reduce the severity of inflammation-related long-term consequences for affected patients and may avoid unnecessary revision surgery.

## Introduction

Arthroplasty is a successful and nowadays essential surgery. In patients suffering from end-stage joint destruction due to osteoarthritis or secondary to rheumatoid arthritis (RA) or other conditions, it significantly improves quality of life by reducing pain, restoring function, and increasing physical activity. Rather infrequently, metals released from implant materials cause local and systemic complications (1).

Peri-implant osteolysis is a highly prevalent non-infectious local complication and known to be driven by chronic innate immune responses to wear particles, whereas the pathogenesis of rarely occurring acute or chronic inflammation due to delayed type hypersensitivity (DTH) is mediated by an adaptive immune response (2). In early-onset osteolysis both processes are described to be tightly intertwined (3). Classical symptoms of an acute periprosthetic joint inflammation are most commonly related to bacteria induced periprosthetic joint infection (PJI). However, after microbiological exclusion of septic complications, the differential diagnosis of sterile metal-related hypersensitivity remains conceivable (4). Multiple factors influence the outcome of the inflammatory processes and their biological consequences, such as elemental composition and chemical speciation of wear debris, exposure level and exposure duration as well as the local environment (5, 6). Materials used in arthroplasty hold the potential to induce DTH reactions. In particular, metals such as nickel, cobalt and chromium are attributed to DTH (7), whereas the chemical speciation and protein interaction of these metals determine their immunogenicity (8, 9). Metal ions and particles released due to corrosion processes are linked to adaptive immune responses in arthroplasty (10). Titanium-, cobalt- and chromium-containing particles were detected in periprosthetic compartments with cobalt and chromium present in the non-particulate/ionic state (11). Beside metal ions, other compounds used in arthroplasty like initiators of the polymerization of methyl methacrylate (bone cement) are known sensitizers (12). Antibiotic additives of bone cement are also known to have sensitization capacity (13).

Metal ions and other sensitizing compounds have the potential to bind self-peptides or self-proteins and form haptens (14). In cutaneous and drug-associated DTH these haptens can act as antigens and, upon subsequent exposure, cause clonal expansion of specific effector memory T cells and clinical manifestation of acute inflammation. Comparable mechanisms, characterized by perivascular lymphocytic infiltrate, have been identified in the peri-implant membrane (15). DTH reactions beyond the peri-implant membrane in the adjacent bone marrow are currently proposed and discussed (16).

Generally, local hypersensitivity reactions to orthopedic implants are difficult to diagnose, mainly because of considerable individuality of patients' inflammation and associated characteristics as well as due to close similarities with PJI in clinical presentation. Sterile toxicity induced hypersensitivity reactions and other reasons for joint inflammation like RA additionally hinder the recognition of DTH based on clinical examination. Reliable diagnostic tools and algorithms are urgently needed to ensure the diagnosis of implant-related DTH. This report illustrates a possible step-wise diagnostic work-up for cases of suspected peri-implant hypersensitivity.

## Case Presentation

**Figure 1:**
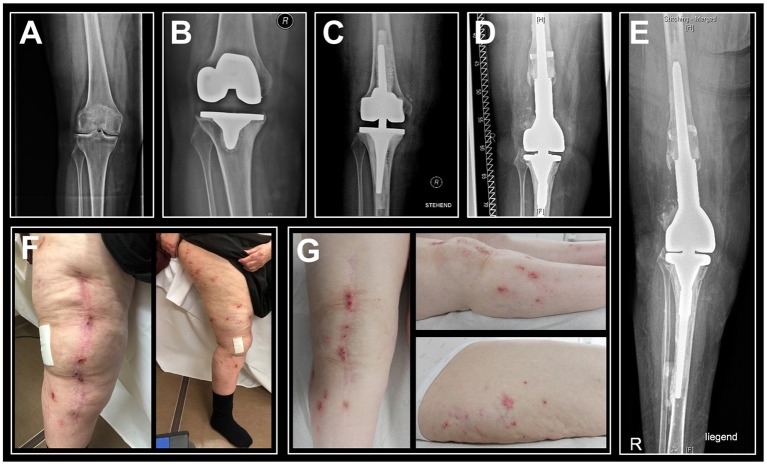
Sequential anteroposterior radiographs of the patient's right knee indicate progression of bone loss and photographs of the right leg show persisting eczema. **(A)** Radiography prior to primary knee replacement depicts joint degeneration secondary to rheumatoid arthritis, 2009/03. **(B)** Pre-revisional radiograph indicates peri-implant bone loss and loosening of the tibial component, 2012/03. **(C)** Post-revisional radiograph depicts intense intraoperative usage of cement and progressive bone loss, 2012/07. **(D)** Radiographic status at the time of hypersensitivity diagnostics, 03/2018. **(E)** Latest radiographic status (patient in supine position), 01/2019. **(F)** Eczema at the time of hypersensitivity diagnostics, 03/2018. **(G)** Follow-up examination in the aftermath of rituximab therapy indicated an unimproved cutaneous status, 03/2019.

We report the case of a 71-year-old female patient with chronic RA who presented at our institution 4 years after right-sided re-re-revision total knee arthroplasty with debilitating chronic pain, swelling, and severely itchy eczema (2014/03–2018/03) (Figure 1). This patient has a complex medical and orthopedic history (Supplementary Table 1). She was diagnosed with seropositive RA in 1969. As this chronic inflammatory, joint damaging disease progressed she underwent therapy with different disease-modifying anti-rheumatic drugs, extensive physiotherapy and eventually several surgical procedures addressing her initially dominating right-sided hand and wrist pain. Since 2005, the patient had been suffering from progressive bilateral knee pain, culminating in near immobilization in 2009. Considering clear right-sided domination of clinical complaints, functional deficit and radiographic lesions characteristic for RA-induced joint destruction, she underwent right-sided cemented primary total knee arthroplasty in 2009/05. Both intra- and short-term postoperative courses were uneventful. At routine follow-up 6 months postoperatively, the patient showed excellent joint function and overall mobilization, minimal swelling, a non-irritated surgical scar and denied relevant pain of the right knee.

However, 33 months after primary implantation (2012/02), the patient presented at our institution with right-sided knee pain, moderate local swelling, local hyperthermia, distinct itchy eczema scattered across the right leg and involving the surgical scar. She reported a progression of those symptoms over the previous 12 months. Ad interim, she had received steroid injections from her treating rheumatologist twice, both of which alleviated her complaints for about 10 weeks. However, in the course of that consultation, routine blood tests and knee joint aspiration were performed to rule out infection. Blood leukocyte count was normal, CRP was 37 mg/l (Ref., <5 mg/l) and synovial leukocyte count was 352/μl. Aspiration had not yielded sufficient volume to run microbiological cultures. Further, standard radiographs revealed loosening of the tibial component. Considering that the patient had a history of Lichen ruber, involving predominantly the trunk and her lower legs, dermatologists were consulted to evaluate the skin lesions, and judged them as pruritic dermatosis not associated to Lichen ruber.

The synopsis of findings led to the suspicion of a low-grade PJI and thus to two-stage revision arthroplasty (2012/04, 2012/07). Here, bone cement without antibiotic additive was used due to the patient's report of a previous allergic reaction to gentamicin. Histopathological examination of tissue samples taken during explanation of the primary implant and implantation of the secondary implant endorsed a wear particle induced type periprosthetic membrane and a low-grade infection, respectively. Microbiological analysis of cultures of synovial fluid, periprosthetic tissue and sonication fluid revealed no significant growth. However, the patient underwent empiric antibiotic therapy. Yet, the postoperative course was characterized by persistent pain and swelling, and eczema progression. Due to suspicion of persisting PJI, she underwent further arthrocenteses in 2012/09 and 2013/08, both of which showed no significant microbial growth. Of note, the femoral component of the secondary endoprosthesis implanted after temporary arthrodesis in 2012/07 was composed of cobalt-chromium-molybdenum (CoCrMo) and high nitrogen stainless steel with a nickel content of approximately 10% (Supplementary Table 2).

Since the patient's complaints aggravated further she sought a second opinion at a different hospital. There, the secondary implant was revised in 2014/02, following microbial detection of multi-resistant *Staphylococcus epidermidis* (according to the discharge letter; the respective microbiology report was not available). In the consecutive year, she underwent another two-stage revision due to suspected persistent low-grade infection of the right knee (2015/06, 2015/08). However, there was no documentation of relevant microbial growth and histopathology revealed a type 3 periprosthetic membrane (i.e., a combination of a wear and an infection induced histology pattern) (17). As in the primary and the secondary implants, both, tibial, and femoral components of the tertiary and of the quaternary implants contained the CoCrMo alloy (Supplementary Table 2). After the third revision arthroplasty, the patient's complaints improved moderately for roughly 8 weeks before deteriorating to the maximum of reported pain, skin irritation, and immobilization in 2018. Then, she was referred to us by rheumatologists to rule out PJI of the right knee again. At that consultation, the patient reported that she had been diagnosed with cobalt allergy through skin patch testing in 2015. Considering the patient's progressive eczema surrounding and including her arthroplasty scars, the dermatologists' judgement of that not being associated with the pre-existent Lichen ruber, and the patient's report of metal allergy, acute inflammation due to implant-related metal hypersensitivity seemed more probable.

Therefore, we sampled periprosthetic fluid via percutaneous arthrocentesis for microbiological, pathological, and cytological examinations. Furthermore, multi-element analyses of synovial fluid and whole blood were performed to investigate relevant metal quantities. Serum and whole blood of the patient were obtained for a lymphocyte transformation test (LTT), which includes exposure to all potentially sensitizing metals and substances used in joint arthroplasty. The cellular composition of whole blood and synovial fluid were analyzed with flow cytometry and specific focus on T cells and its subsets. The findings of those tests are presented and discussed below.

Of note, in 2019/01 the patient presented at the department of rheumatology of our institution where she underwent inpatient rituximab therapy and multimodal RA staging. Again, systemic infection-indicating parameters (CRP, leukocytes) were within reference levels and analysis of the right knee's synovial fluid did not indicate infection. At the time of the most recent orthopedic follow-up examination (2019/03), recovery had not been reached.

The patient's medical and implant data including information on manufacturers, compositions and fixation methods of the knee arthroplasty implants are concisely listed in Supplementary Tables 1, 2.

## Multi-Stage Diagnostics

Routine blood tests showed non-elevated systemic levels of CRP (4.8 mg/l; Ref., < 5 mg/l) and leukocytes (6.7/nl; Ref., 3.9/nl−10.5/nl). Flow cytometry analysis of the peri-implant synovial fluid sampled by percutaneous arthrocentesis revealed a leukocyte count of 1,105/μl; 36.2% of total leukocytes were found to be mononuclear cells and 63.8% polymorphonuclear (PMN) cells. In accordance with clinical guideline, leukocyte numbers of >2,000/μl and the presence of >70% PMNs of the leukocytes would match the criteria for PJI (18). The examination of the periprosthetic joint fluid by a surgical pathologist revealed a low cell content and no signs of infection which is indicative for the absence of a bacterial high-grade infection. Moreover, no gout or pseudogout crystals were found. In summary, cytological and pathological examinations proved no evidence for bacterial infection. However, results from microbiological examination were pending at the time of the above mentioned diagnostics.

**Figure 2:**
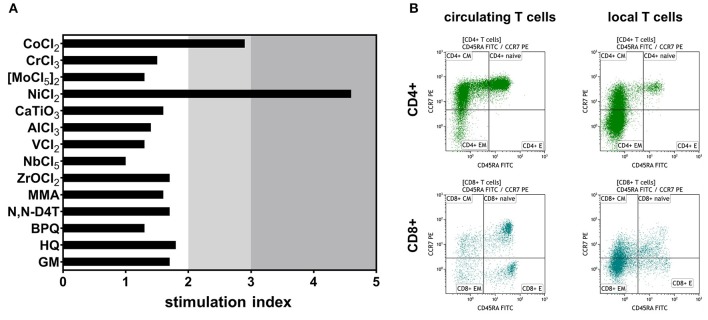
Lymphocyte transformation test (LTT) and T cell subsets analyses point toward T cell mediated hypersensitivity as the root cause for local inflammation. **(A)** Exposure to various arthroplasty-relevant noxae revealed increased T cell proliferation following cobalt and nickel exposure. Mean stimulation index (proliferation with antigen divided by proliferation without antigen) values (*n* = 3) of >2 indicate marginal sensitization and values >3 can be considered positive. **(B)** Scatter dot plots of circulating T helper cells (CD4+) and cytotoxic T cells (CD8+) indicate abundance of effector memory T cells in the synovium of the affected knee. MMA, methyl methacrylate; N,N-D4T, N,N-dimethyl-p-toluidine; BPQ, benzoyl peroxide; HQ, hydroquinone; GM, gentamicin.

LTT was performed as previously shown (19), with modifications toward endoprostheses-relevant allergens. It revealed a moderate cellular sensitization in the sense of a DTH reaction to nickel by a stimulation index of 4.6 and marginally to cobalt by a stimulation index of 2.9, indicating circulating allergen specific T cells (Figure 2A). There were no significant signs of sensitization following exposure to any other tested relevant metals, cement monomers or antibiotics. The used salts and compounds are depicted in Supplementary Table 3.

Blood multi-element analysis with inductively coupled plasma mass spectrometry (ICP-MS) showed moderately, yet increased systemic cobalt (2.10 μg/l) and chromium (0.94 μg/l) levels (Table 1). With 44.2 μg/l cobalt and 12.7 μg/l chromium in the peri-implant synovial fluid, local metal levels clearly surpassed systemic ones. Thus, metal level quantification hinted at the potential of released cobalt and chromium to promote inflammatory symptoms, whereas a contribution of cobalt can be confirmed considering the LTT and the previously performed patch test.

**Table 1:** Systemic and local metal levels [μg/l] analyzed with inductively coupled plasma mass spectrometry.

	**Co**	**Cr**	**Mo**	**Ni**	**Ti**	**Al**	**Nb**	**V**	**Zr**
Whole blood at presentation	2.1 (+)	0.94 (+)	0.7	0.5	14.9	<10.0	<2.0	<0.20	<2.0
Whole blood at follow-up	2.7 (+)	0.69 (+)	0.5	1.6	9.5	<10.0	<2.0	<0.20	<2.0
Reference values^*^ (whole blood)	<1.21	0.14–0.52	0.3–1.3	<3.8	<16.1	<11.4	<2.0	<0.20	<2.0
Synovial fluid at presentation	44.2	12.7	1.8	<1.0	6.3	<20	<0.20	0.20	<1.0

+, increased;

* *, reference values were established by metal level quantification in >6,000 individual blood samples; limits of quantification are reported in Supplementary Table 4*.

Fluorescence-activated flow cytometry analysis of T cell subsets showed 13.1% CD3+ T cells in the peri-implant synovial fluid within the total population of CD45+ cells, the majority of T cells being effector memory T cells (Table 2). In particular, 66.5% of all CD4+ and 51.2% of all CD8+ T cells belonged to the effector memory compartment, which represents an 8-fold increase in CD4+ T cells and a 4-fold increase in CD8+ T cells when compared to peripheral blood levels (Figure 2B). Up to 31.2% of the CD4+ T cell population were shown to be regulatory T cells, which is an 8-fold increase vs. circulating T cell numbers (Table 1). In summary, flow cytometry analysis identified local invasion or expansion of effector memory and regulatory T cells. This is typical for DTH and pathogen induced immune reactions.

**Table 2:** Systemic and local T cell subsets.

**T cell basic subsets**				
Marker	CD45+	CD3+	CD4+	CD8+
Gate	Singlets	CD45+	CD3+	CD3+
Subset	Leukocytes	T cells	Th cells	Tc cells
Blood [% gate]	65.7	20.6	83.1	14.9
Synovial fluid [% gate]	50.6	13.1	66.6	24.3
Fold change [local/systemic]	0.8	0.6	0.8	1.6
**Th subsets**				
Marker	CCR7+/CD45RA+	CCR7-/CD45RA-	CCR7-/CD45RA+	
Gate	CD4+	CD4+	CD4+	
Subset	Naïve	Effector memory	TEMRA	
Blood [% gate]	39.3	8.2	*n* <50	
Synovial fluid [% gate]	2.6	66.5	*n* <50	
Fold change [local/systemic]	0.1	8.1	—	
**Tc subsets**				
Marker	CCR7+/CD45RA+	CCR7-/CD45RA-	CCR7-/CD45RA+	
Gate	CD8+	CD8+	CD8+	
Subset	Naïve	Effector memory	TEMRA	
Blood [% gate]	45.5	12.5	25.0	
Synovial fluid [% gate]	17.5	51.2	5.0	
Fold change [local/systemic]	0.4	4.1	0.2	
**Treg subsets**				
Marker	CD25+/CD45RA+	CD25+/CD127-	CD25+/FoxP3+	
Gate	CD4+	CD4+	CD4+	
Subset	Naïve	Treg	Treg	
Blood [% gate]	*n* <50	4.1	4.5	
Synovial fluid [% gate]	*n* <50	31.2	21.6	
Fold change [local/systemic]	—	7.8	4.8	

However, microbiological examination displayed no signs of bacterial growth following aerobic and anaerobic incubation for seven and 14 days, respectively. Therefore, a bacterial peri-prosthetic infection as underlying reason for the inflammatory symptoms was excluded.

Except for the T cell subset analysis with flow cytometry, the presented methods were in line with standard diagnostic methods that are routinely performed by accredited medical testing laboratories and therefore readily available for most hospitals in industrialized countries. Flow cytometry analysis of T cell subsets was performed by commercially available dry pre-formulated antibody panels (DURAClone, Beckman Coulter) as previously shown for blood samples (20).

## Discussion

We report the case of a patient with a long-standing history of RA who has been suffering from chronic pain syndrome following right-sided total knee arthroplasty for many years. Generally, RA is not considered to be a risk factor for poor patient reported outcome in knee arthroplasty (21). Yet, on suspicion of bacterial low-grade infection the reported patient's artificial knee joint had been revised three times. At the time of performance of the majority of the diagnostic tests, the painful knee joint was found to be sterile. Since primary implantation the patient has suffered severe bone loss surrounding the implant. Despite previously diagnosed hypersensitivity to cobalt by patch testing, a CoCrMo containing implant was used in the course of the latest revision surgery. Summing up the results of the performed multi-stage diagnostics, we conclude that the patient's symptoms are most likely associated to aseptic peri-implant inflammation due to metal release from the artificial joint and cobalt hypersensitivity. Whether cutaneous alterations of the lower extremities are hypersensitivity-induced cannot be answered by the performed diagnostics. Following the expertise of the involved dermatologists the eczema was most likely a pruritic dermatosis not associated to pre-existent autoimmune Lichen ruber. Considering its onset after primary TKA, metal hypersensitivity represents a potential trigger.

The patient suffers from concomitant rheumatic disease, which is known to show abundant numbers of effector T cells in the synovium (22). It cannot be excluded that these local cells derive from her rheumatoid comorbidity. At the time of DTH diagnostics, the patient's rheumatic disease was not treated pharmacologically. However, 9 months after hypersensitivity diagnostics, she underwent inpatient immunosuppressive therapy with rituximab in the course of the treatment of chronic rheumatoid polyarthritis (elsewhere). This treatment was indicated, since there were no signs of local bacterial infection and previous treatment with rituximab was well-tolerated.

Rituximab acts as a B cell depleting agent. Follow-up clinical examination after 10 weeks of inpatient therapy showed that the symptomatology of the aseptic peri-implant inflammation and cutaneous alterations had not significantly improved. The acute inflammatory phase of DTH is known to be initially driven by clonal expansion of effector T cells. Yet, in the course of RA treatment classical methotrexate therapy, which would additionally target T cell activation and T cell receptor expression, had not been appropriate due to the patient's reported methotrexate intolerance. Leflunomide or a combination of leflunomide with TNF inhibitors could be considered as potential treatment for patients with RA. In a case of non-DTH induced implant-related inflammatory arthritis, long-term prednisolone and leflunomide therapy were reported to control the symptoms (23). In the presented case, however, the eczema of the lower extremity worsened despite three months of leflunomide monotherapie. Other immunosuppressive, cell-therapeutic and surgical treatment options must be discussed. In terms of immunosuppressive therapy, a major aim would be targeting both, RA and metal hypersensitivity. Another option for pharmaceutical treatment targeting the suppression of T cell immunity are calcineurin inhibitors like cyclosporine. This drug is known for successful prevention of transplant rejection; another T cell mediated reaction, closely resembling a DTH reaction. Cyclosporine in combination with TNF inhibitors is administered for RA treatment in hepatitis C virus positive patients (24). In the future, a regulatory T cell-mediated cell therapy as currently used in the prevention of transplant rejection may be advised (25). In terms of surgical intervention, treatment options are not as diverse. Due to chronic inflammation of the artificial joint, the patient is at risk of further bone loss and decreased bone quality. CoCrMo-free knee endoprostheses large enough for treatment of such advanced bone loss, e.g. CoCrMo-free tumor prostheses, are currently not available on the market. Arthrodesis must be considered, as it may be performed without the use of CoCrMo-containing components and may allow for extremity salvage. However, surgical options are critically limited not only by the apparent intolerance to metal implant components and the patient's allergic predisposition but also by the actual gap between remaining bones. In fact, operative tracks to achieve symptom relief and retain function are nearly exhausted. To “surgically terminate” metal exposure, the associated local inflammation and further local and systemic adverse effects, ultima ratio would be amputation.

Facing the depicted drastic course of routine joint replacement vigorously illustrates the need for advanced metal hypersensitivity diagnostics and their integration into clinical decision making. Recently, another diagnostic algorithm related to the one suggested in this report, has been proposed (26). An important part of that algorithm is the initial exclusion of PJI and mechanical complications. Diagnostic tools to rule out PJI more rapidly than microbiological examination are currently under development (27). Furthermore, systemic and local metal levels should be quantified to evaluate metal release potentially inducing DTH reactions and inflammation. This analysis should include all relevant elements contained in the individual implant set-up. Subsequent examination of putative DTH is important, in particular before revision surgery. The two options currently available to diagnose DTH comprise the patch test for contact allergy and LTT. In arthroplasty, the prognostic and diagnostic value of these methods is called into question on the basis of controversial results of clinical studies (28). One reason for inconclusive results could be that the systemic immune status does not sufficiently reflect the periprosthetic immune cell composition (16). It is also possible that the spectrum of locally released potential allergens in terms of physicochemical diversity is more distinct than displayable in the LTT by soluble salts. To improve future DTH diagnostics, we envision to extend the LTT by stimulation with endogenously generated haptens and by T cell analysis of the synovial fluid (e.g., by flow cytometry).

Further characterization of local T cell subsets would be of value to strengthen LTT results. In particular, modern methods for analyzing the T cell receptor repertoire could help detect clonal expansion of effector memory T cells (29). Secondly, we envision to optimize the LTT by using cell depleted artificial joint aspirates as antigen cocktails comprising the endogenously produced haptens of the individual patient. The latter measure is aimed to mimic closely endogenous conditions in DTH inflamed joints. These changes and additions to the current LTT protocol may yield a diagnostic tool of superior sensitivity, still excluding the risk of iatrogenic sensitization inferred by the patch test. Future studies evaluating these diagnostic innovations may help to trace potential links between locally present allergens and known metalorganic compounds, breaking new ground in the development of novel biomarkers for DTH. This could be accomplished through methods readily used in proteomics.

It is of uttermost importance to establish suitable thresholds for specific T cell subpopulations in synovial fluid and other local compartments, to perform local T cell analyses in cohorts of sufficient sample size and to finally combine those with systemic and local metal exposure determination and advanced LTT. This proceeding may be useful to back the clinical diagnosis of implant-related hypersensitivity in the future. An ideal diagnostic tool for DTH in arthroplasty must not be influenced in sensitivity and specificity by concomitant infection or any other reason for joint inflammation.

## Concluding Remarks

This case demonstrates the importance of a thorough diagnostic work-up and suggests an algorithm for consecutive testing of patients with indications or merely anecdotal evidence of DTH to their implant material. This algorithm consists of exclusion of PJI, local and systemic metal level quantification, an LTT specific for metals and organic substances used in arthroplasty and local and systemic T cell subset analyses. Additionally, we recommend establishing thresholds for defining clinically relevant quantities of effector memory T cell and regulatory T cell fractions. This could help to derive unequivocal diagnosis of implant-related hypersensitivity and to facilitate the identification of therapeutic needs of patients suffering from aseptic periprosthetic inflammation in the aftermath of joint replacement surgery. Even though hypersensitivity is rare, patients scheduled to undergo primary arthroplasty should be informed about severe and possibly devastating consequences including loss of ambulation, amputation, and severe systemic adverse events.

The presented complex case underlines that the diagnosis of hypersensitivity to wear and corrosion products from endoprostheses can currently not be substantiated but only be considered after the exclusion of septic complications. In summary, there is a distinct clinical need for specific implant-related hypersensitivity diagnostics. Close collaboration between clinicians, toxicologists, immunologists, and routine medical laboratories is required.

## Data Availability Statement

The datasets generated for this study are available on request to the corresponding author.

## Ethics Statement

Written informed consent has been obtained from the patient for publication of this case report.

## Author Contributions

AR and JS developed the conceptual idea to the manuscript and wrote the manuscript. AR took clinically care of the patient, performed the clinical examinations, and read up on clinical history of the patient. MO and JS performed the flow cytometry analysis. MO and SG performed quantitative analyses of the flow cytometry data. KH performed the metal level quantification and hypersensitivity testing. All authors reviewed and edited the manuscript.

### Conflict of Interest

The authors declare that the research was conducted in the absence of any commercial or financial relationships that could be construed as a potential conflict of interest.
