# Supplementary material for: Diagnosis of Metal Hypersensitivity in Total Knee Arthroplasty: A Case Report
Source: Front Immunol. 2019 Nov 27;10:2758. doi: 10.3389/fimmu.2019.02758 (PMC6890602; doi:10.3389/fimmu.2019.02758)
Supplement: Supplementary file 1 [file Table_1.DOCX]

**SUPPLEMENTARY TABLE 1 |** Patient’s medical baseline data and concomitants

| **Baseline data** |  |
| --- | --- |
| Sex | female |
| Age at primary knee arthroplasty | 62 years |
| Age at revision of primary knee endoprosthesis | 65 years |
| Age at revision of secondary knee endoprosthesis | 67 years |
| Age at revision of tertiary knee endoprosthesis | 68 years |
| Age at presentation (DTH diagnostics) | 71 years |
| Age at most recent follow-up | 72 years |
| BMI at presentation (DTH diagnostics) | 31 kg/m² |
| **Concomitant diseases at presentation** | |
| Seropositive rheumatoid arthritis | |
| Complex regional pain syndrome (focus: right leg) | |
| Arterial hypertension | |
| Sensory axonal polyneuropathy | |
| Low-grade mitral insufficiency | |
| Aortic Valve Sclerosis | |
| Drug-induced osteoporosis | |
| History of Hepatitis C (viral load last detectable 2016/12) | |
| Lichen ruber exanthemicus | |
| Latent Tuberculosis infection | |
| Esophageal hiatal hernia Type I | |
| Gastroesophageal reflux disease (GERD) | |
| Tilidine abuse | |
| History of basal skull fracture (2011) | |
| History of removal of “cold” thyroid nodule (1973) | |
| **Known hypersensitivities reported at presentation** (DTH diagnostics) | |
| Clindamycin | |
| Gentamicin | |
| Cobalt | |
| Linezolid | |
| Zolpidem | |
| Teriparatide | |
| Rifampin | |
| Isoniazid | |
| Methotrexate | |
| Leflunomid | |
| Plaster | |
| **Concomitant medication at presentation** (DTH diagnostics) | |
| Bisoprolol | |
| Amlodipine | |
| Torasemide | |
| Valsartan | |
| Levothyroxine | |
| Omeprazole | |
| Clonazepam | |
| Tilidine & Naloxone | |
| Thiamine, Pyridoxine & Cyanocobalamin | |
| **Concomitant medication at most recent follow-up (2019/03)** | |
| Bisoprolol hemifumarat | |
| Amlodipine | |
| Torasemide | |
| Valsartan | |
| Doxazosin | |
| Levothyroxine | |
| Moxonidine | |
| Omeprazole | |
| Clonazepam | |
| Tilidine & Naloxone | |
| Thiamine, Pyridoxine & Cyanocobalamin | |

* Abbreviations: BMI, body mass index; DTH, delayed type hypersensitivity

**SUPPLEMENTARY TABLE 2 |** Patient’s orthopedic history

| **Time scale** | **Indication for Orthopedic Intervention** | **Orthopedic Intervention with Implant Data According to Manufacturer** |
| --- | --- | --- |
| 1969 | initial diagnosis of **seropositive rheumatoid arthritis** |  |
| 1985 | RA-induced destruction of MCPJ II-V of the right hand | synovectomy of MCPJ II-V |
| 1986 | RA-induced destruction of PIPJ II-V of the right hand | synovectomy of PIPJ II-V |
| 1998 / 10 | RA-induced destruction of the right elbow joint | synovectomy of right elbow joint and right-sided radial resection |
| 1999/01 | RA-induced destruction of wrist joint, right>left | right-sided ulnar head resection and Mannerfelt's total wrist fusion |
| 2002/10 | RA-induced destruction of MCPJ II-V of the right hand | implantation of silastic Swanson’s implants in MCPJ II, III, IV, V of right hand |
| 2002/10 | peri-implant infection MCPJ II-V of the right hand | removal of Swanson’s implants from MCPJ II-IV of the right hand, lavage, debridement, antimicrobial therapy |
| 2003/08 | pain of MCPJ II-V and PIPJ II-V of the right hand | surgical revision of MCPJ II-V including resection of metacarpal heads II-V, Littler-release of PIPJ II-V of the right hand |
| 2009/05 | RA-induced knee joint destruction, right > left | **cemented primary total knee arthroplasty**  (DePuy Orthopaedics Inc., P.F.C.® SIGMA® Knee System; CS Femoral Component Sz. 3, RT, non-porous, 66 mm M/L, 61 mm A/P; Tibial Tray, cemented, Sz. 2.5, Tibial Insert (RP-STAB) rotating platform 15 mm / Sz. 3; Oval Dome Patella, 38 mm) |
| 2012/04 | suspected PJI of the right knee joint | **explantation of primary implant due to suspected PJI & temporary arthrodesis using an intramedullary stabilized PMMA spacer** |
| 2012/07 | temporary arthrodesis of the right knee joint | **cemented revision total knee arthroplasty**  (Smith & Nephew, RT-PLUSTM, Solution; Femoral Component, CoCrMo, FeCrNiMnMoNbN, UHMWPE, Sz. 6; Tibial Component, CoCrMo, Sz. 4; C-Plug, Sz. 14 mm; Tibial Insert, Ti6Al4V, UHMWPE Size 4 / 14 mm) |
| 2014/02 | suspected PJI of the right knee joint | **explantation of revision implant** due to suspected PJI **& temporary arthrodesis using an intramedullary stabilized PMMA spacer** |
| 2014/03 | temporary arthrodesis of the right knee joint | cemented revision total knee arthroplasty (LINK; SN 131015/1651; SN 131216/0672; SN 130820/0767; REF 15-8522/42, CoCrMo, conus 12/14mm, L= 160 mm, K= 14 mm, A= 9 mm); REF 16-2853/32, CoCrMo, Sz. S; REF 16-2840/02, CoCrMo, UHMWPE, Sz. S; REF 16-2817/02, CoCrMo, UHMWPE, Sz. S) |
| 2015/06 | suspected PJI of the right knee joint | **explantation of revision implant** due to suspected **& temporary arthrodesis using an intramedullary stabilized PMMA spacer** |
| 2015/08 | temporary arthrodesis of the right knee joint | cemented revision total knee arthroplasty (LINK; SN 141208/3402; SN 140408/2909; SN 140805/2656; REF 15-8526/40, CoCrMo, conus 10/12 mm, L=130 mm, K= 12 mm; A= 8 mm; REF 15-8522/06, TILASTAN® Ti6Al4V, CoCrMo, conus 12/14 mm, L=50mm; REF 15-8523/44, CoCrMo, conus 12/14 mm, L= 130 mm, K= 6mm, A = 12 mm);) REF 162853/21, CoCrMo, UHMWPE, Sz. S; REF 16-2817/32, CoCrMo, UHMWPE, Sz. S; REF 16-2840/02, CoCrMo, UHMWPE, Sz. S) |

Abbreviations: CoCrMo, cobalt-chromium-molybdenum alloy; FeCrNiMnMoNbN, iron-chromium-nickel-manganese-molybdenum-niobium-nitrogen alloy; MCPJ, metacarpophalangeal joints; PIPJ, proximal interphalangeal joints; PJI, periprosthetic joint infection; PMMA, polymethylmethacrylate; RA, rheumatoid arthritis; Ti6Al4V, titanium-aluminum-vanadium alloy; UHMWPE, ultra-high-molecular-weight polyethylene

**SUPPLEMENTARY TABLE 3 |** Salts and compounds used for the individual stimulation procedures in the lymphocyte transformation test (LTT).

| salt, compound | CAS number |
| --- | --- |
| Cobalt(II) chloride | 7791-13-1 |
| Chromium(III) chloride | 10060-12-5 |
| Molybdenum(V) chloride | 10241-05-1 |
| Nickel(II) chloride | 7791-20-0 |
| Calcium titanate | 12049-50-2 |
| Aluminum(III) chloride | 7446-70-0 |
| Vanadium(II) chloride | 10580-52-6 |
| Niobium(V) chloride | 10026-12-7 |
| Zirconium(IV) oxide chloride | 13520-92-8 |
| Methyl methacrylate | 80-62-6 |
| N,N-Dimethyl-p-toluidine | 99-97-8 |
| Benzoyl peroxide | 94-36-0 |
| Hydroquinone | 123-31-9 |
| Gentamicin | 1405-41-0 |

**SUPPLEMENTARY TABLE 4 |** Limits of quantification [µg/l] for inductively coupled plasma mass spectrometry (ICP-MS) of the individual metals quantified in whole blood and synovial fluid.

| matrix | Co | Cr | Mo | Ni | Ti | Al | Nb | V | Zr |
| --- | --- | --- | --- | --- | --- | --- | --- | --- | --- |
| whole blood | 0.02 | 0.10 | 0.20 | 0.20 | 2.0 | 10.0 | 2.0 | 0.20 | 2.0 |
| synovial fluid | 0.20 | 2.0 | 0.20 | 1.0 | 2.0 | 20.0 | 0.20 | 0.20 | 1.0 |
